# New Partially Water-Soluble Feedstocks for Additive Manufacturing of Ti6Al4V Parts by Material Extrusion

**DOI:** 10.3390/ma16083162

**Published:** 2023-04-17

**Authors:** Ralf Eickhoff, Steffen Antusch, Dorit Nötzel, Thomas Hanemann

**Affiliations:** 1 Institute for Applied Materials, Karlsruhe Institute of Technology, Hermann-von-Helmholtz-Platz 1, D-76344 Eggenstein-Leopoldshafen, Germany; 2 Department of Microsystems Engineering, University Freiburg, Georges-Koehler-Allee 102, D-79110 Freiburg, Germany

**Keywords:** material extrusion, FFF, FDM, FFD, additive manufacturing, titanium alloys, Ti6Al4V, water-soluble binder, feedstock

## Abstract

In this work, a process chain for the realization of dense Ti6Al4V parts via different material extrusion methods will be introduced applying eco-friendly partially water-soluble binder systems. In continuation of earlier research, polyethylene glycol (PEG) as a low molecular weight binder component was combined either with poly(vinylbutyral) (PVB) or with poly(methylmethacrylat) (PMMA) as a high molecular weight polymer and investigated with respect to their usability in FFF and FFD. The additional investigation of different surfactants’ impact on the rheological behaviour applying shear and oscillation rheology allowed for a final solid Ti6Al4V content of 60 vol%, which is sufficient to achieve after printing, debinding and thermal densification parts with densities better than 99% of the theoretical value. The requirements for usage in medical applications according to ASTM F2885-17 can be fulfilled depending on the processing conditions.

## 1. Introduction

Additive manufacturing, quite often also denoted as 3D printing, is nowadays an established fabrication method of polymer, metal and ceramic parts. In most cases, 3D printing is used for rapid prototyping, but these methods have gained more and more importance for rapid tooling and even rapid manufacturing of small-scale series [1,2,3,4,5,6,7,8]. Due to their history of invention and the operation principle, the most important techniques are VAT photopolymerization such as stereolithography (SLA), material extrusion (MEX) such as fused filament fabrication (FFF), and powder bed fusion such as selective laser melting (SLM) or electron beam melting (EBM). In recent years, a pronounced material and process development widened the initial material limitations and enabled the adaptation of SLA and MEX-based printing techniques for the processing of ceramic and metal materials. FFF and related techniques benefit from the long feedstock development experience in powder injection molding (PIM) for the fabrication of dense and warpage-free metal and ceramic parts according to the following process chain [9]:

1. Feedstock compounding of using suitable binders, additives and a huge solid load (ceramics: ≥50 vol%; metals: ≥60 vol%) considering spherical fillers ideally;
2. Printing via MEX techniques;
3. Debinding, often a combination of liquid pre-debinding with thermal debinding;
4. Sintering with conditions depending on the filler’s properties.

With respect to the knowledge gained from PIM, several groups were able to realize different ceramics and metals applying MEX methods [9,10,11,12,13,14,15,16,17,18,19]. Perhaps due to the more complex sintering step, only a few reports dealing with FFF of metals were published [20,21,22,23,24,25,26]. Currently, lightweight and medical applications are upcoming fields of interest, especially using titanium alloys such as Ti6Al4V. The application of the latter as an implant material is outlined in the ASTM F2885-17 standard, describing, amongst others, the relevant mechanical properties [27]. The additive manufacturing applying Ti6Al4V via powder-based methods such as SLS, SLM or EBM has been reported and is commercialized partially [28,29,30,31]; quite recently, MEX methods were also used for part realization [32,33,34,35,36].

A comprehensive investigation of the feedstock flow behaviour is essential to meet the two contradictory requirements of a suitable feedstock. On the one hand, the solid load must be as high as possible to minimize shrinkage and warpage risk during sintering. On the other hand, the solid load must not be too high to allow a good melt flow behaviour, enabling a defect-free extrusion and filament deposition without additional voids due to solid overfilling. A highly filled feedstock with a solid particle loading represents a complex system with the occurrence of elastic (by the solid) and viscous (by the polymer) properties in the molten state. With respect to a proper description of the melt flow behaviour, it is necessary to measure in addition to the shear rate dependent viscosity the viscoelastic flow via oscillation measurements applying a rotary rheometer. The latter technique was exercised for different feedstock systems [17,18,37], and quite recently in [36]. It is possible to determine the complex shear modulus G* containing the contributions of the storage modulus G’ (elastic part) and the loss modulus G’’ (viscous part) (1). With the knowledge of the applied angular frequency, the complex viscosity can be estimated (2). The direct relationship between the complex shear modulus and the complex viscosity is shown in (3) [38]. An accurate determination of the viscoelastic flow and the feedstocks yield point is of particular importance for feedstock and printing parameter development with respect to proper and defect-free deposition of a freshly printed layer on the previous one. A more detailed description of the relevance of the complex shear modulus G’’ can be found in [36] and especially in [38].

G* = G’ + iG’’(1)

(2) $$\eta *=\eta ’+i\eta ’’=\frac{G’}{\omega }+i\;\left(-\frac{G’’}{\omega }\right)$$

(3) $$\left|\eta^{*}\right|=\left|\frac{G^{*}}{\omega }\right|$$

The following work extends the research on suitable feedstock systems for Ti6Al4V from the initially used nonpolar wax/polyethylene (PE) mixtures to polar and therefore partially water-soluble binder systems, which simplifies especially the solvent-based pre-debinding towards eco-friendly solvents such as water, instead of polluting hexane/heptane solvents [39].

## 2. Materials and Methods

### 2.1. Polar Feedstock Development

With respect to potential applications in health care products such as implants and in continuation of previous published work [36], Ti6Al4V (Grade 23FE, Heraeus, Hanau, Germany) was selected as a metal filler [40]. Due to the demanding mechanical property requirements in medical engineering, this grade shows a low carbon and oxygen content according to the ASTM F2885-17 standard [27], derived from the specifications for metal injection molding (MIM) produced Ti6Al4V surgical implants.

**Figure 1:**
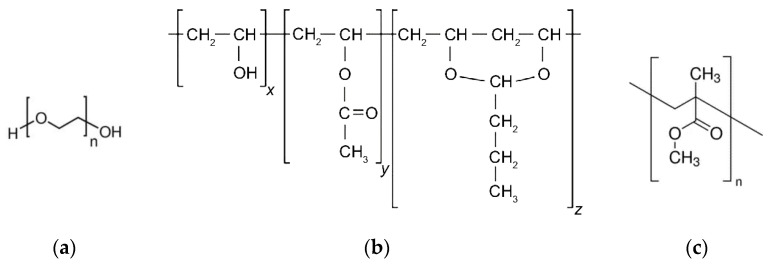
Chemical structures of the used main binder components: (**a**) polyethylene glycol (PEG); (**b**) poly(vinylbutyral) (PVB); (**c**) poly(methylmethacrylate) (PMMA).

In significant contrast to previously published work [36], two polar binder systems consisting of the low molecular weight polyethylene glycol (PEG) and the high molecular weight polymers poly(vinylbutyral) (PVB) or poly(methylmethacrylate) (PMMA) have been investigated (Figure 1). Due to synthesis conditions, PVB can be described as a copolymer containing the aspired butyral group, an acetyl- as well as the highly polar hydroxy functionality. Both binders, PEG/PVB and PEG/PMMA, have been successfully applied for ceramics in FFF and injection molding, enabling crack-free and dense alumina or zirconia parts [12,13,41,42,43,44]. In this work, PEG with different average molar weights M_W_ (2000, 4000, 6000, 8000, 20,000 g/mol) (C. Roth GmbH Co KG, Karlsruhe, Germany) have been applied. PVB Mowital B30H (Kuraray Europe GmbH, Frankfurt, Germany) and PMMA Degalan G7E (Roehm GmbH, Darmstadt, Germany) were selected as high molecular weight polymers. Stearic acid (SA, C. Roth GmbH Co KG, Karlsruhe, Germany) as well as two commercial additives, PAT-77/P and PAT-659/CB (E. & P. Wuertz GmbH Co. KG, Bingen, Germany), with proprietary composition, recommended by the vendor for polymer/metal feedstocks, have been used as surfactants. Normally, the surfactant amount is directly related to the specific surface area of the filler, but due to the low specific surface area (SSA) of the used Ti6Al4V, a fixed surfactant concentration of 5 wt% of the whole binder amount is applied and subtracted from the low molecular weight PEG. The thermal behaviour of all investigated organic binder components can be found in Table 1.

In addition to the data submitted by the vendors, a comprehensive educt characterization is mandatory, especially in the case of the selected Ti6 Al4V filler. With respect to feedstock development, the following four main particle properties must be characterized:

- Particle size distribution;
- Specific surface area;
- Particle density;
- Particle morphology.

The particle size distribution was measured via laser diffraction (LA-950 Horiba Ltd., Kyoto, Japan), the specific surface area (SSA) by the Brunauer-Emmett-Teller adsorption method (BET, Gemini VII 2390, Micromeritics Instr. Corp., Norcross, GA, USA), the particle density by helium pycnometry (Pycnomatic ATC, Porotec, Germany) and, finally, the particle morphology with a SEM Supra 55 (Zeiss, Oberkochen, Germany).

### 2.2. Feedstock Preparation

A torque recording mixer-kneader (W50-EHT, Brabender GmbH, Duisburg, Germany) was applied for the new polar feedstock compounding. The blade’s rotating speed was set to 30 rpm, which is equivalent to a maximum shear rate of 36 1/s for 1 h compounding time. The compounding temperature was set to 110 °C (PEG/PVB) and 160–170 °C (PEG/PMMA) according to the different glass transition temperatures of the high molecular weight polymer. Further details can be found in [36].

### 2.3. Rheological Characterization

All feedstock systems were investigated by two common established rheometric methods. The temperature and shear rate dependent viscosity were measured by a high-pressure capillary rheometer (Rheograph 25, Goettfert Werkstoff-Pruefmaschinen GmbH, Buchen, Germany). For better comparison with other feedstock systems [36], identical measuring parameters (temperature: 160 °C; capillary diameter and length: 1 and 30 mm, shear rate range: 1 to 5000 1/s) were used. With respect to the determination of the complex flow behaviour, especially the detection of potential yield points as a function of the feedstock composition, oscillatory measurements in the amplitude sweep (AS) mode applying a rotary rheometer (Gemini HR Nano, Netzsch-Geraetebau GmbH & Co., Selb, Germany) equipped with a plate-plate geometry (20 mm diameter, gap 1 mm, smooth surface) were applied. To be consistent with previously reported work, identical measuring parameters (temperatures, frequency, shear stress range) were used [36].

### 2.4. Material Extrusion (MEX)

#### 2.4.1. Fused Filament Fabrication (FFF)

After feedstock compounding and pelletizing with an impact mill (Granulator 1514, Rapid Germany, Kleinostheim, Germany), the granules were reshaped into filaments by a single screw filament extruder (Noztek pro HT, Noztek, Shoreham, UK). All FFF-printing trials were performed with a German RepRap X350pro printer (Feldkirchen, Germany) applying different extruder nozzles (0.15, 0.3 and 0.4 mm). With respect to acceptable printing results, some machine modifications were necessary; details can be found in [36].

#### 2.4.2. Fused Feedstock Deposition (FFD)

In the case of FFD printers, pellets or granules can be used instead of filaments. The two-component FFD printer FFD 150H (3d-figo GmbH, Salzkotten, Germany) carries two small extruders placed directly above the printheads. In all cases, a nozzle diameter of 0.4 mm was used. For better material adhesion and printed part removal, the printing platform was covered with PE-coated spring steel. It was necessary to preheat the feedstock pellets in the extruder to accelerate the extrusion process. Two different slicers for machine control were applied: Ultimaker Cura and Repetier-Host (V 2.2.2).

### 2.5. Debinding, Sintering and Hot Isostatic Pressing

The debinding step was subdivided into two parts. Initially, the PEG was removed in water (40 °C, 24 h), which generates open pores. For this, the printed parts were placed on a die plate in a glass reactor setup. Then, thermal debinding was conducted prior to sintering in a metal sinter furnace (MUT Advanced Heating GmbH, Jena, Germany) with a maximum debinding temperature of 550 °C, subsequently followed by sintering (max. temperature 1350 °C). The thermal treatment was performed under argon atmosphere to avoid further oxygen contamination. In the case of selected samples and with respect to enhanced density values, hot isostatic pressing (HIP, HIP3000, Dieffenbacher GmbH, Eppingen, Germany) was applied.

### 2.6. Characterization of Sintered Samples

A set of different techniques were used for a comprehensive sample characterization covering density measurements following the Archimedes principle (Secura 225D-1S equipped with YDK 01, Sartorius Lab Instruments GmbH & Co KG, Göttingen, Germany) and metallographic preparation described in detail in [36]. With respect to the final carbon and oxygen content in the sintered part, a combustion analysis (CS600 and TC600, LECO Instruments GmbH, Mönchengladbach, Germany) was performed.

## 3. Results and Discussion

### 3.1. Material Characterization

From previous work dealing with powder injection molding, FFF or FFD in the case of ceramic or metal part fabrication, a huge solid load is necessary to achieve dense parts (ceramic: >99% theoretical density, metals: >98% theoretical density). The presence of spherical particles is mandatory to obtain moderate viscosity values. The morphology of the applied Ti6Al4V powder is spherical with a monomodal distribution around 30 µm, a density around 4.4 g/cm³ and a SSA value of 0.15 m²/g [36], which is quite small in contrast to established ceramic materials such as alumina or zirconia.

### 3.2. Feedstock Compounding

#### 3.2.1. PEG/PVB Binder System

**Figure 2:**
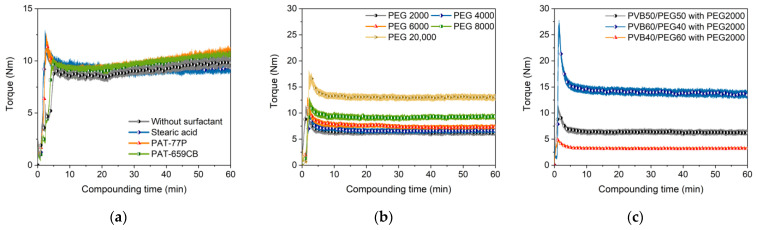
Compounding of PEG/PVB-based binder (110 °C, 60 vol%) with different compositions. (**a**) PEG 8000 as low molecular component and with or without different surfactants (5 wt%); (**b**) variation of the PEG M_W_ with 5 wt% SA as surfactant; (**c**) variation of PEG/PVB ratio applying PEG 2000 as low molecular binder part and 5 wt% SA as surfactant.

PEG/PVB binder systems in combination with alumina or zirconia fillers and SA as a surfactant were previously used in ceramic injection molding [41,42,43] and in FFF [12,13]. Here, the influence of three different surfactants on compounding has been investigated. Figure 2a shows the time-depending compounding torque of the PEG/PVB mixtures without and with the three different investigated surfactants applying a PEG M_W_ of 8000 g/mol. In general, the compounding curve can be split in three phases, namely the filling phase, the mixing phase and, finally, the equilibrium phase [45]. From the torque vs. time diagram the quality of the resulted feedstock can be evaluated, e.g., a constant final equilibrium torque value is a strong hint for a homogenous feedstock composition [45]. The observed compounding behaviour is slightly different from the previously investigated wax/PE mixtures [36]. The applied solid load of 60 vol% yielded a low maximum torque value of around 12 Nm (kneaded at 110 °C), which is almost twice the value of the related wax/PE mixtures (kneaded at 125 °C) [36]. After passing the mixing stage, a pronounced torque drop can be seen followed by a slight increase in all cases; only the usage of SA delivers an almost constant torque value. The compounding behaviour was almost identical with and without a surfactant. The addition of the two PAT surfactants caused a slight torque increase. The negligible surfactant influence can be attributed to the very small SSA and the spherical particle shape. In the literature, some other binder systems were employed for Ti6Al4V as a solid filler:

- Unknown proprietary binder with solid load of 59 vol% [33,34];
- Polyethylene vinyl acetate/poly(propylene ethylene)/polyisobutene with SA as a surfactant [35], solid load 60–65 vol%;
- Poly(propylene ethylene)/maleic acid anhydride functionalized polypropylene with a solid load up to 60 vol% [37].

The average molecular weight of the used PEGs had a clear impact on the compounding behaviour. While the equilibrium torque values of the low molecular weight PEG (2000, 4000, 6000, 8000 g/mol) were close together, a further increase to 20,000 g/mol caused almost a torque doubling (Figure 2b). This can be attributed to the increasing probability of chain entanglement accompanied by increasing inner friction. The variation of the PEG/PVB ratio can also be used for viscosity adjustment. Applying PEG 2000 as low molecular weight binder part, the increase of the PEG 2000 fraction yielded a pronounced torque drop and a faster access to the equilibrium state (Figure 2c).

#### 3.2.2. PEG/PMMA Binder System

**Figure 3:**
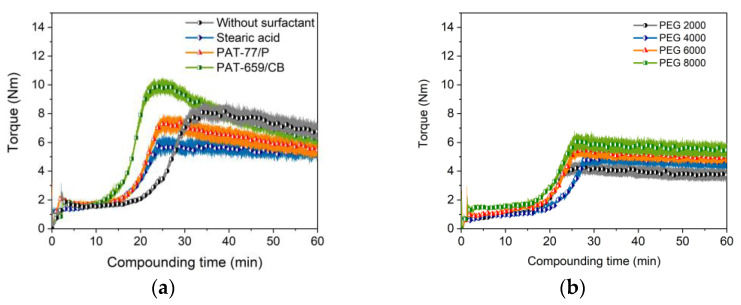
Compounding of PEG/PMMA-based binder (170 °C, 60 vol%). (**a**) PEG 8000 as low molecular component and with or without different surfactants (5 wt%) at a 1 to 1 PEG/PMMA ratio; (**b**) variation of the PEG M_W_ (5 wt% SA).

PEG/PMMA systems have been also introduced as a binder in PIM and material extrusion. The influence of three PMMAs with different M_W_ and granule morphology on compounding, melt rheology and micro powder injection molding of zirconia and the final ceramic density was described in [44,46]. A binder, consisting of PEG (M_W_: 1500 g/mol) and a PMMA emulsion with SA as a surfactant, was used in metal injection molding of tungsten carbide/cobalt mixtures [47]. The liquid debinding behaviour of PEG/PMMA feedstocks in water with Ti6Al4V as a filler and different SA contents is described in [48]. The binder possesses a huge excess of PEG (M_W_: 4000 g/mol) relative to PMMA of almost 9:1, simplifying the dissolution process [48]. Hayat et al. investigated the influence of the PEG M_W_ (1500–20,000 g/mol) on the flow and water debinding behaviour of titanium-filled PEG/PMMA feedstocks for metal injection molding [49]. The higher the average molecular weight, a higher viscosity and slower solvent debinding in water can be observed [49]. As in the previous case, the influence of the three different surfactants (5 wt%) on the compounding process were investigated; again, the surfactant replaced partially the PEG moiety. Figure 3a presents the torque as function of time and used surfactant (PEG 8000). Due to the huge glass transition temperature of the PMMA (96–109 °C), a compounding temperature of 170 °C was necessary. The compounding curve (Figure 3a) looks quite different than in the PEG/PVB system shown in Figure 2a and can be attributed to the shape of the PMMA pellets and the higher glass transition temperature. Prior to mixing, the pellets have to be liquified, which can be seen in the pronounced extension of the filling and mixing phase up to 30 min instead of 5 min in the PEG/PVB binder. The influence of the surfactants is more obvious; the PAT-659/CB especially caused a significant initial torque increase. After 1 hour compounding time, only the addition of SA delivered a constant torque value. The M_W_ of the used PEGs had only a small impact on the compounding behaviour (Figure 3b). As expected, an increasing M_W_ caused a slight torque increase; the compounding process is more dominated by the pellet melting.

**Figure 4:**
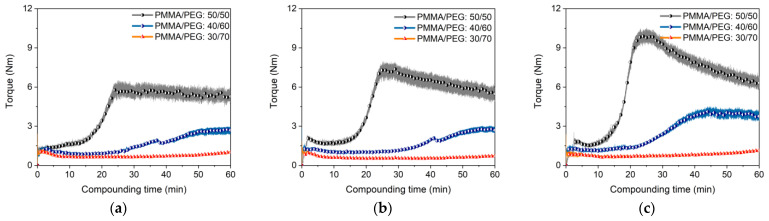
Compounding using different surfactants (5 wt%). (**a**) SA; (**b**) PAT-77P; (**c**) PAT-659/CB.

**Figure 5:**
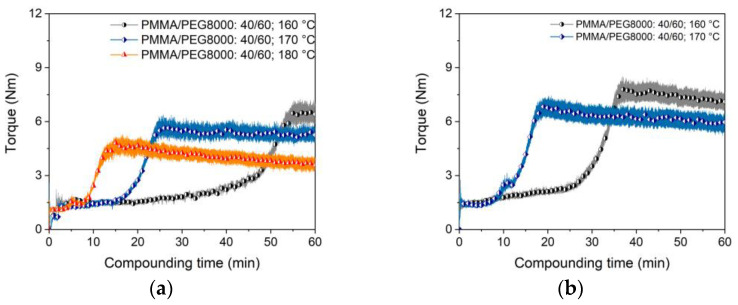
Compounding of PEG/PMMA-based binder and SA as surfactant. (**a**) PMMA/PEG 8000 ratio 40/60 at different temperatures (solid load 60 vol%); (**b**) PMMA/PEG 8000 ratio 40/60 at different temperatures (solid load 65 vol%).

Following Table 1, a compounding temperature of 170 °C is quite high and, e.g., very close to the decomposition temperature of SA. Therefore, a feedstock processing at lower temperatures is recommended, which can be achieved by viscosity reduction due to an increase of the binder amount with lower M_W_ [49]. Figure 4a–c show for the different surfactants the change of the torque with proceeding compounding time and variable PMMA to PEG (here PEG 8000) ratio. In all cases, a pronounced maximum torque drop can be observed, the impact of the PMMA liquification shrinks with the reduction in the PMMA amount and the low viscosity of the PEG 8000 dominates. The differences between the surfactants diminishes as well with higher PEG concentrations. Therefore, the PEG amount increase allows for a compounding temperature reduction or for higher solid loadings. Both effects—the temperature drop and solid load increase—are depicted in Figure 5. Figure 5a shows the influence of the temperature on compounding applying a feedstock with a PMMA/PEG8000 ratio of 40/60 and a solid load of 60 vol% Ti6Al4V. A higher compounding temperature yields in a lower torque as well as in a faster equilibrium stage. At a higher load of 65 vol% (Figure 5b), a temperature increase helps in the same manner, but under consideration of an enhanced SA decomposition.

### 3.3. Rheological Characterization

#### 3.3.1. PEG/PVB Binder with Constant PEG/PVB Ratio

**Figure 6:**
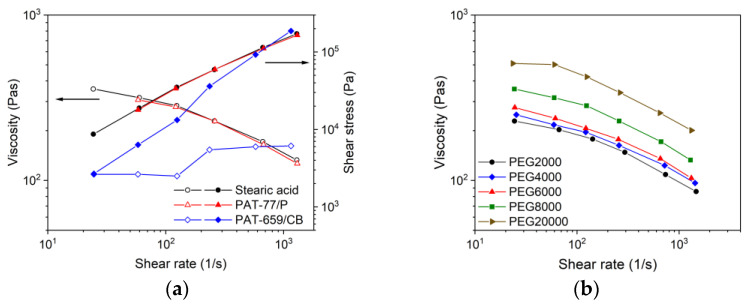
(**a**) Shear rate dependent melt viscosity as well as shear stress at 160 °C of PEG8000/PVB-based binder at a solid load of 60 vol% and different surfactants; (**b**) variation of the PEGs’ average molecular weight with 5 wt% SA as surfactant.

As described previously [36], a comprehensive description of the feedstocks’ melt flow behaviour is crucial for the successful usage in FFF, FFD as well as in injection molding. Figure 6a shows for the mixture PEG/PVB and different surfactants the melt flow behaviour at a solid load of 60 vol% and a temperature of 160 °C. A viscosity measurement without surfactant was not possible due to phase separation at moderate and higher shear rates. Whilst the presence of SA and PAT-77/P caused the typical pseudoplastic (shear-thinning) flow with almost identical viscosity values, the addition of PAT659/CB resulted in an unusual melt flow with a viscosity increase at larger shear rates representing slight dilatancy. This phenomenon was reproducible. Dilatancy normally occurs if a superstructure with increasing attractive forces is formed and should be attributed here to the molecular structure of the surfactant, which is unfortunately proprietary. The viscosities were comparable to the ones applying wax/LDPE feedstocks at the same solid load and identical measuring temperature [36]. The variation of the PEG M_W_ had an impact on the resulting viscosity in that increasing M_W_ yielded a viscosity increase retaining the pronounced pseudoplastic flow, which is favourable for injection molding (Figure 6b). The usage of PEGs with a M_W_ of at least 6000–8000 g/mol is due to the higher softening temperature advantageous for FFF, FFD and injection molding enabling a higher greenbody stability.

**Figure 7:**
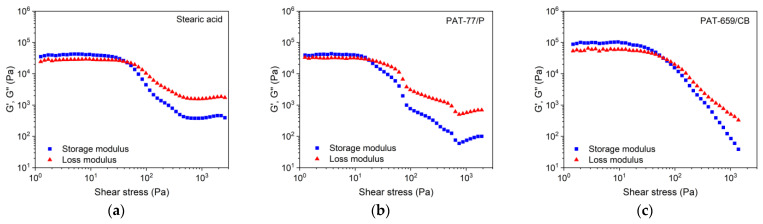
Storage (G’) and loss modulus (G’’) of PEG 8000/PVB systems containing three different surfactants (temperature 160 °C, solid load 60 vol%) via oscillatory test applying amplitude sweep. (**a**) SA; (**b**) PAT-77/P; (**c**) PAT-659/CB.

**Figure 8:**
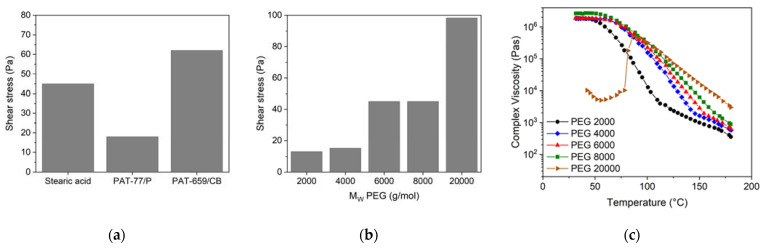
Yield points for PEG/PVB feedstocks (160 °C, solid load 60 vol%). (**a**) Applying PEG 8000 and different surfactants; (**b**) applying PEGs with different M_W_ and SA as surfactant; (**c**) complex viscosity as function of temperature and PEG with different M_W_ and SA as surfactant.

A molten feedstock represents a complex mixture of polar and nonpolar materials and the simultaneous presence of solid and liquid matter causing a viscoelastic flow behaviour, which can be investigated by oscillation rheology measuring the storage G’ and the loss modulus G’’ separately, representing the solid-state elastic behaviour (G’) as well as the liquid flow (G’’) (Figure 7). The investigated feedstocks containing different surfactants show at low shear stresses a larger storage than loss modulus describing the dominance of the more elastic part of the complex modulus. At a certain shear stress, the two curves cross and the loss modulus dominates, representing the viscous part. This crossover is denominated as the yield or flow point, which is of particular importance for FFF or FFD printing. SA (Figure 7a) shows the smallest complex moduli values of all investigated feedstocks; PAT-77P is at low shear stresses close to SA (Figure 7b), and PAT-659/CB (Figure 7c) possesses the highest complex moduli values. For better comparison, Figure 8a compares the yield point values at 160 °C for the investigated PEG8000/PVB feedstocks with different surfactants. In the case of SA as additive, Figure 8b demonstrates the influence of the PEG M_W_ on the yield point. Increasing M_W_ causes a pronounced yield point value raise due to the large possibility of chain entanglement causing enlarged inner friction. These results were supported by a temperature sweep investigation, where a PEG Mw increase from 2000 to 20,000 g/mol yielded a strong complex viscosity increase especially at higher temperatures by more than one decade (Figure 8c).

#### 3.3.2. PEG/PVB Binder with Different PEG/PVB Ratios

**Figure 9:**
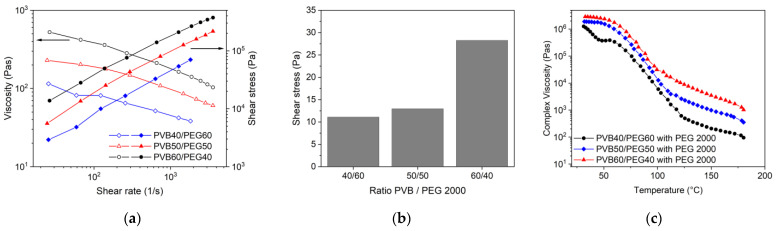
(**a**) Melt viscosity of PEG 2000/PVB feedstocks with different PEG/PVB ratios (160 °C, solid load 60 vol%); (**b**) yield point dependence on PEG2000/PVB ratio; (**c**) complex viscosity as function of temperature and PEG 2000/PVB ratio.

Following the results obtained from compounding, it can be expected that the variation of the PEG/PVB ratio has a pronounced impact on the melt rheology. Figure 9a describes the change of the melt flow as a function of shear rate and PEG2000/PVB ratio. Decreasing PVB amounts allowed a clear viscosity reduction retaining the pseudoplastic flow. The yield point is also affected by the ratio of the low and high molecular binder moiety. Whilst the decrease of a 50%:50% ratio of PEG 2000 to PVB down to 40% PVB has only a small impact on the yield point, the increase up to 60% PVB results in a significant yield point gain by almost a factor of 2.5 (Figure 9b). The variation of the PEG 2000/PVB ratio has an influence on the complex viscosity change with increasing temperature, especially at higher temperatures passing the softening temperatures of PEG 2000 (52 °C) and PVB (68 °C) (Figure 9c).

#### 3.3.3. PEG/PMMA Binder with Constant PEG/PMMA Ratio

**Figure 10:**
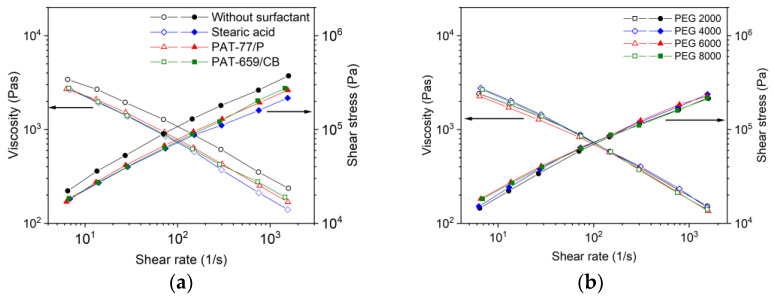
(**a**) Shear rate dependent melt viscosity as well as shear stress at 160 °C of PEG8000/PMMA-based binder (solid load 60 vol%) and different surfactants; (**b**) variation of the PEG M_W_ with 5 wt% SA as surfactant (solid load 60 vol%, 160 °C).

In contrast to the related PEG/PVB feedstocks at the same solid load, the melt viscosity is almost higher by one decade, which can be attributed mainly to the huge average molecular weight of the used PMMA (M_W_ PMMA~159,000 g/mol; PVB~35,000 g/mol, Table 1). The presence of any of the surfactants delivered a small viscosity drop relative to the feedstock without surfactant; the variation of the surfactant type itself did not show any relevant melt flow discrepancy (Figure 10a). The variation of the PEG M_W_ did not alter the resulting viscosity; remarkably, all systems possessed a typical pseudoplastic flow (Figure 10b).

**Figure 11:**
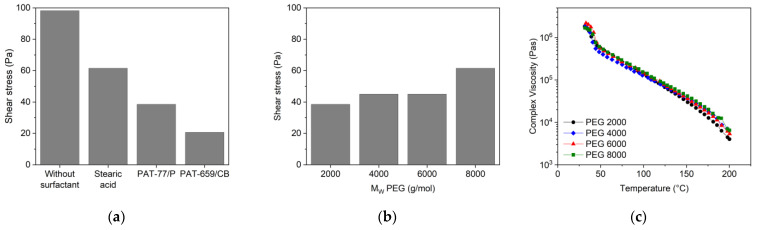
Shear stress values of the yield points for PEG/PMMA feedstocks (160 °C, solid load 60 vol%). (**a**) Applying PEG 8000 and different surfactants; (**b**) applying PEGs with different M_W_ and SA as surfactant; (**c**) complex viscosity as function of temperature and PEG with different M_W_ and SA as surfactant.

As in the PEG/PVB feedstocks, a yield point could be detected by the measurement of the complex viscosity depending on the used surfactant and PEG M_W_ (Figure 11a,b). All applied surfactants lowered the shear stress value for the yield point in contrast to the feedstock without surfactant (Figure 11a); the two PATs especially had a pronounced impact. The substitution of the PEG 8000 by PEGs with smaller M_W_ caused a reduction of the yield point shear stress value (Figure 11b). The influence of M_W_ on the complex viscosity is negligible as well (Figure 11c). These results are in strong contrast to the above presented PEG/PVB feedstocks (Figure 8) and should be attributed to the huge PMMA M_W_.

#### 3.3.4. PEG/PMMA Binder with Different PEG/PMMA Ratios

**Figure 12:**
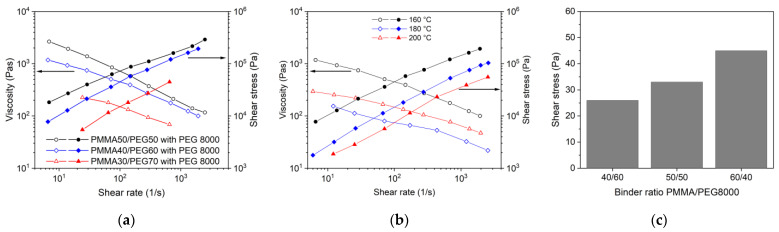
(**a**) Change of melt flow with increasing PEG 8000 moiety in the binder (solid load; 60 vol%, T = 160 °C, SA as surfactant); (**b**) temperature influence on a PEG60/PMMA40 feedstock applying PEG 8000 (solid load 60 vol%, SA as surfactant); (**c**) variation of the PEG 8000/PMMA ratio and resulting shear stress change for the yield point (SA as surfactant).

In addition to the previously described PEG M_W_ variation, the flow behaviour can be adjusted by the change of the PEG/PMMA ratio. Increasing PEG 8000 amounts caused a remarkable viscosity drop at constant temperature, especially at concentrations beyond 60% (Figure 12a). A temperature gain from 160 °C up to 200 °C for a system PMMA40/PEG60 (PEG 8000) also enabled a viscosity drop (Figure 12b). The PEG/PMMA ratio variation towards larger PMMA amounts resulted in a yield point shear stress value increase (Figure 12c).

#### 3.3.5. Solid Load Variation

**Figure 13:**
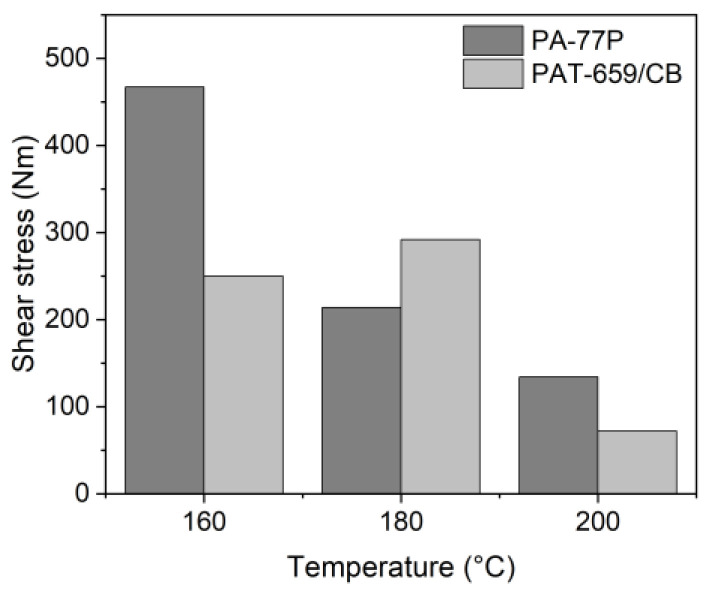
Shear stress values for the yield points for PEG60/PMMA40 (PEG 8000) applying the surfactants PAT-77P and PAT-659/CB at different temperatures (solid load 65 vol%).

Within the PEG/PVB feedstock composition, it was not possible to realize systems with solid loads beyond 60 vol% in an acceptable quality suitable for FFF or FFD printing. With the experience previously gained by the variation of used PEG, the PEG/PMMA ratio, selected surfactant and exploiting the temperature dependence of the melt viscosity, it was possible to realize feedstocks with an enhanced solid load of 65 vol% by an increase of the PEG 8000 moiety. Figure 13 shows the shear stress values for the yield point applying the two PAT surfactants at different temperatures and a solid load of 65 vol%. The values at 160 °C are significantly higher (almost a factor of 10) than the related ones at a solid load of 60 vol%. In previously described results, e.g., in Figure 11, PAT-77P possessed a higher yield point shear stress value than the PAT-659/CB surfactant; the unexpected behaviour at 180 °C may be attributed to phase separation. It has to be noted that the solid load increase from 60 to 65 vol% hampered the feedstock preparation and measurement significantly.

### 3.4. Material Extrusion (MEX)

#### 3.4.1. Processing of PEG/PVB Feedstocks

Following the results from rheological characterization, three different PEG/PVB systems with a Ti6Al4V load of 60 vol% and varying M_W_ of PEG were selected for filament fabrication applying the Noztek filament extruder, equipped with a 2.8 mm nozzle; Table 2 summarizes the achieved filament parameters. All extruded filaments did not show any extrudate swelling directly behind the nozzle. Therefore, the filament diameters are close to the required 2.85–3 mm filament diameter requested by the modified X350Pro FFF printer. This molten filament behaviour is in significant contrast to the previously used wax/LDPE feedstocks [36]. The surface appearance of the filaments was not unique: whilst the PEG 2000 containing filament showed a smooth surface, the two others possessed a rough surface, which may be attributed to phase separation between PEG and PVB and a deteriorated particle wetting.

**Figure 14:**
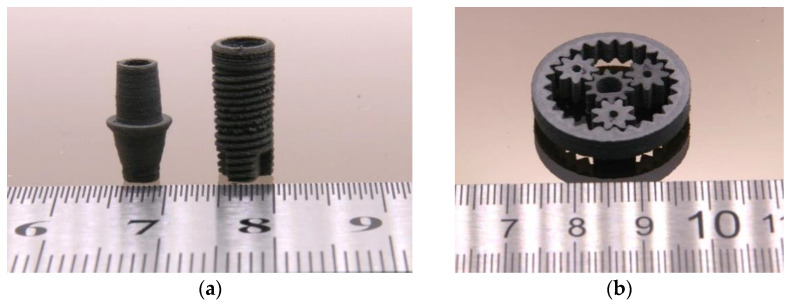
FFF-printed PEG 20,000/PVB parts. (**a**) Dental implant; (**b**) planetary gear train.

As in previous work, the filament extrusion delivered rigid filaments which cannot be winded; hence, about 50 cm long straight filaments were extruded on a metal cooling track [36]. These new filaments were then stored in a vacuum oven at 47 °C not less than 24 h to avoid any water adsorption, which is typical for feedstocks with the capability of hydrogen bridge formation, especially PVB [50]. After drying, the filament rods were introduced into the printer’s filament extruder from above. The FFF process itself was hampered by the pronounced phase separation at the used printing temperature around 200 °C, especially at low PEG average molecular masses. Table 3 lists the best FFF printing parameters for the different PEG/PVB mixtures applying in all cases SA as a surfactant. Only in the case of the PEG 20,000 the phase separation at low temperatures could be suppressed and devices printed (Figure 14). With the exception of the printing temperature, the printing parameters are almost identical to the previously reported wax/PE feedstocks [36]. Due to the huge viscosity under FFF printing conditions, it was not possible to print parts applying a feedstock with 65 vol%.

Due to the pronounced phase separation, printing via FFD was impossible according to the higher shear forces at elevated temperatures.

#### 3.4.2. Processing of PEG/PMMA Feedstocks

Because of the comprehensive rheological investigation on the PEG/PMMA feedstocks, the system PEG60/PMMA40 with PEG 8000 as low molecular binder component was selected for FFF printing trials after filament extrusion. Table 4 summarizes the processing parameters; unfortunately, in the case of the PAT659/CB-containing mixture, a straight feedstock track could not be prepared. As in the PEG/PVB systems, an extrudate swelling after passing the nozzle was not observed. The feedstocks with the two different PAT surfactants were suitable for FFF (PAT77-P) and FFD (PAT77-P, PAT659/CB) printing. Surprisingly, SA could not be used due to the phase separation at the printing temperature, which is almost like PEG/PVB feedstocks. It seems to be that at higher temperatures the solubility of SA in PEG/PMMA feedstocks is reduced. Table 5 lists the best printing parameters. In the case of FFD, the printing as well as the platform temperatures are slightly increased in comparison to the ones in FFF, which is attributed to a better adhesion of the first layer on the printing platform. Directly after printing, the parts showed a low greenbody stability, which is caused by the platform temperature close to the softening temperature of the used binder component PEG 8000 (Table 1). At lower temperatures, a pronounced stiffness increase could be observed. The reduced mechanical stability at temperatures around 60 °C and higher is a drawback for the FFD method, because the softened material tended to clog the feedstocks’ granule hopper located above the extruder due to ascending heat.

**Figure 15:**
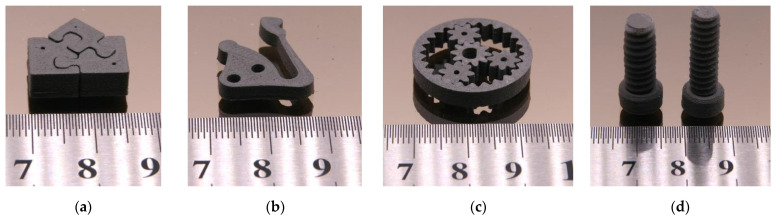
Printed PEG (8000)60/PMMA40 feedstock (solid load 60 vol%). (**a**) Puzzle (FFF); (**b**) test structure with long cantilever and holes [18]; (**c**) planetary gear train (FFF); (**d**) screws (FFD).

Both printing methods—FFF and FFD—allowed the printing of small parts in a good and reliable quality, but the surface appearance of the FFD-printed parts was slightly worse than the FFF-printed ones, which can be attributed to the not yet optimized printhead. Figure 15a–c show FFF-printed parts with different demanding geometries, Figure 15d via FFD-printed screws. Due to the huge viscosity in FFF and FFD printing conditions, especially the process related small shear rates, it was not possible to print parts applying a feedstock with 65 vol%.

### 3.5. Debinding and Sintering

**Figure 16:**
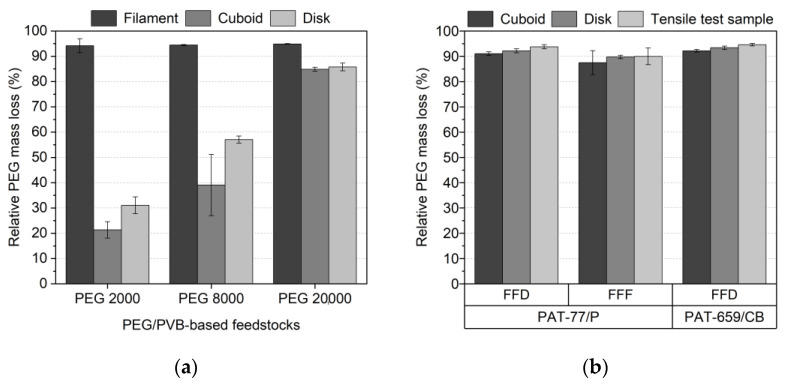
Relative PEG mass loss of water-soluble PEG-containing feedstocks after 24 h. (**a**) PEG50/PVB50-based systems; (**b**) PEG (8000)60/PMMA40-based systems with different surfactants and printing method.

As in the wax/PE-based feedstocks, debinding was undertaken as a two-step process: the liquid pre-debinding (water, 40 °C, 24 h) was followed by a thermal debinding. Figure 16a shows the PEG removal amount for PEG/PVB with different PEG M_W_ (SA as surfactant) and different test samples (filament, cuboid, disk; see Figure 1 in [36]). Quite surprisingly, the filament showed the best PEG elimination in all investigated systems irrespective of the PEG M_W_. Only in the case of the PEG 20,000 can a comparable removal of the PEG in the filament as well as in the printed specimen be observed. This unusual behaviour was described in the literature [51] and can be attributed to an enhanced thermal instability at elevated temperatures of the low molecular mass PEGs. The printed specimen experienced in contrast to the filament an additional extraordinary heat treatment during printing (extrusion temperature: 90 °C; printing temperature: 230 °C), which caused a PEG decomposition to water-insoluble decomposition products [51]. A further increase of the exposition time (48 h) in water did not show a significant PEG removal improvement. Figure 16b shows the PEG off-take for different printed samples applying the binder PEG(8000)60/PMMA40 with suitable PAT surfactants. In contrast to the PEG/PVB feedstocks, a rapid liquid debinding of all investigated samples and mixtures up to 90% of PEG could be observed. An impact of the huge FFF/FFD printing temperature and resulting PEG decomposition could be found. These results agree with previous investigations [51].

**Figure 17:**
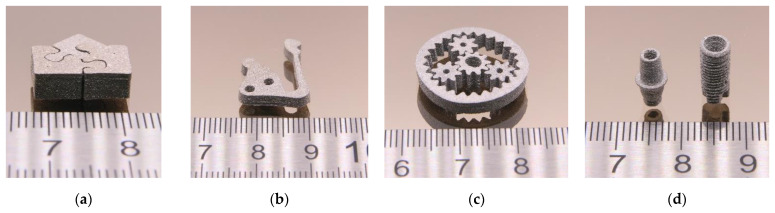
Sintered Ti6Al4V parts (initial solid load: 60 vol%. (**a**) Puzzle (binder PEG/PMMA); (**b**) test structure with long cantilever and holes (binder PEG/PMMA); (**c**) planetary gear train (binder PEG/PVB); (**d**) dental implants (binder PEG/PVB).

Chen et al. also used a similar water-soluble binder system with a Ti6Al4V load of 69.5 vol% [48]. They described a very fast binder removal of almost 100% (sample thickness: 1.5 mm) at 60 °C [48]. Subsequently, after liquid pre-debinding, the samples were thermally debinded followed by a sintering step. Some of the samples were additionally HIPed following ASTM F3001-14 [27] (heating and cooling rate 15 K/min, max. temperature 920 °C for 2 h, max. pressure 100 MPa, argon atmosphere) to achieve higher density values. Figure 17 presents different sintered test structures with various geometric features.

### 3.6. Sinter Part Characterization

#### 3.6.1. Density

**Figure 18:**
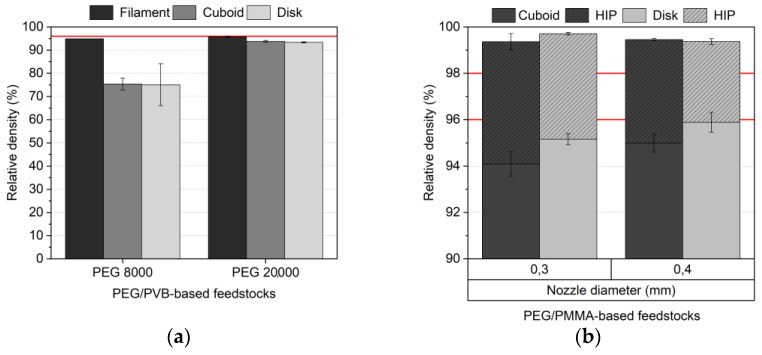
Relative sinter density values (initial solid load 60 vol%) of different FFF-printed test samples: (**a**) PEG/PVB-based feedstocks; (**b**) PEG/PMMA-based feedstocks. Additional densification by HIP marked by hatching. Red lines with respect to values described in ASTM F2884-17 [27].

Being at the end of a process chain, all earlier steps have a pronounced impact on the final density and the resulting mechanical properties. Figure 18a shows the achieved density values for different PEG/PVB mixtures, and Figure 18b for PEG/PMMA systems, both with FFF as the printing method. The red lines in Figure 18 represent the reference value for usage as medical implants with respect to ASTM F2885-17 (without HIP: 96%, with HIP: 98%) [27]. In the case of the PEG/PVB feedstocks, the smaller achievable density of the printed parts in contrast to the filament can be seen again, especially in the case of the PEG 8000 containing mixture (Figure 18a). The usage of PEG 20,000 and the resulting liquid debinding behaviour with the excellent PEG 20,000 removal allowed final density values slightly lower than the value defined by the ASTM F2885-17 value of 96% of the theoretical density. In the case of the PEG/PMMA feedstocks applying the PAT surfactants, the two different nozzle diameters did show a relevant influence on the final sintered part density, because the larger nozzle diameter allowed higher density values between 95% (cuboid) and 96% (disk), which can be attributed to a reduction in the FFF-related void generation during printing (Figure 18b). The additional HIP densification enabled densities higher than the requested 98% up to values close to 100%. It was shown earlier [36] that, e.g., the large infill value of 105% helped to minimize the void generation during printing, allowing huge density values. The use of FFD instead of FFF delivered, in general, lower density values of around 3% percentage points irrespective of which PAT surfactant was used. The same PEG/PMMA feedstock was used in powder injection molding like the wax/PE feedstocks described in [36]. It was possible to achieve densities without HIP around 97% and with HIP close to 100%. These huge values can be explained by the high-pressure injection process and the removal of any voids during cavity mold filling. The achieved density values before and after the HIP process were almost identical to the ones using wax/PE-based feedstocks with a 60 vol% solid Ti6Al4V load as described earlier [36]. Singh et al. [33] reported a best sinter density of 95.6% with an initial solid load of 59 vol% in the applied proprietary binder, which is comparable with the results presented here.

#### 3.6.2. Microstructure

**Figure 19:**
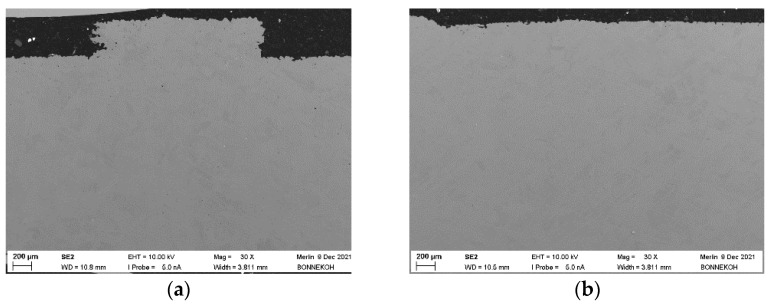
SEM fracture images of sintered parts PEG(8000)60/PMMA40 feedstock (solid load: 60 vol%, 5 wt% PAT-77P). (**a**) sintered (FFF); (**b**) sintered (PIM).

The achievable density depends on the feedstock composition as well as the individual processing steps. Exemplarily, and following the specimen structure described in Figure 1 of [36], Figure 19a,b show the SEM images of a sintered FFF or via PIM fabricated part. The density of the FFF-printed part is 94.7% and the related value for the by PIM fabricated part is 96.9% of the theoretical density, respectively. The number of voids in the case of the FFF-fabricated part slightly increased, which correlates to the lower density value.

#### 3.6.3. Elemental Analysis

**Figure 20:**
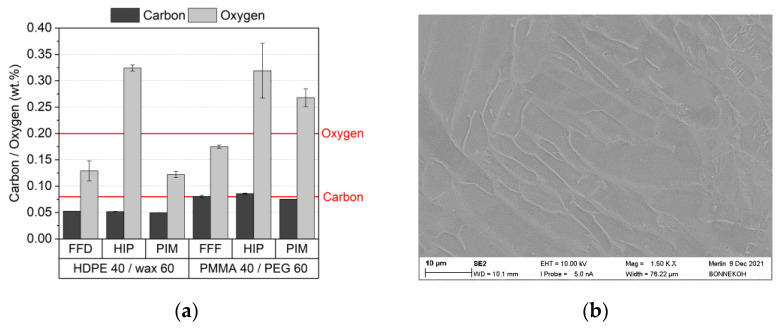
(**a**) Elemental analysis after sintering and HIP applying different replication methods. The red line represents the maximum allowed values according to [27,52]. (**b**) SEM image of sintered and additional HIPed microstructure (initial composition: PEG (8000)60/PMMA40, 5 wt% PAT-77P. solid load 60 vol%, FFF printing, density 99.9%).

The maximum allowed oxygen and carbon content in medical applications is set to 0.2 wt% (oxygen) and 0.08 wt% (carbon) [27,52]. Figure 20a shows for the PEG (8000)60/PMMA40 feedstock the remaining oxygen and carbon concentration values after sintering and partially additional HIP process for FFF and PIM. For better comparison, the previously obtained data applying wax/PE binders were added [36]. The red lines represent the reference values following ASTM F2885-17 [27}. The sintered FFF part fulfils the ASTM criteria for acceptable oxygen and carbon concentration. The HIPed part and the by PIM fabricated part missed the oxygen concentration limit clearly. The wax/PE feedstock, with the exception of the HIPed part, fulfil the oxygen concentration limit. One has to consider that the PEG/PMMA binder system contains a lot of oxygen atoms in the organic molecules, which may increase the remaining oxygen content after thermal processing even under oxygen-free debinding and sintering conditions. The carbon content is not a critical issue. Figure 20b shows a SEM image of the Ti6Al4V microstructure after etching with ammonium hydrogen fluoride, originated from the PEG/PMMA-based feedstock. The typical combined α/β lamellar texture is present, which is in conformity with the requirements defined in [27,52].

## 4. Conclusions and Outlook

It was possible to combine previous work, dealing on the one hand with a partially water-soluble binder system, developed for powder injection molding, and on the other hand the development of FFF and FFD, for the realization of Ti6Al4V parts in good quality almost suitable for use in medical engineering. These water-soluble binders allow an eco-friendly processing by avoiding the harmful hexane in the liquid pre-debinding step of the established wax/PE binder system. A comprehensive rheological investigation on the two binder systems, PEG/PVB and PEG/PMMA, including the change of the PEG´s average molecular weight, enabled the formulation of feedstocks with a solid load of 60 vol%, which were suitable for additive manufacturing of Ti6Al4V parts via FFF and partially for FFD. Liquid pre-debinding in water and thermal debinding as well as sintering metal parts enabled a density close to 96% theoretical density, which is a default value defined by the ASTM F2885-17 for use in medical applications. A further densification by HIP delivered density values better than 99%, fulfilling the ASTM F2885-17.

Future research will focus on the estimation of the mechanical properties as well as on further reduction in the oxygen and carbon content according to ASTM F2885-17.

## Figures and Tables

**Table 1:** Overview of the used binder components (T_S_: softening temperature, T_D_: decomposition temperature).

Component	Density (g/cm³)	M_W_ (g/mol)	T_S_ (°C)	T_D_ (°C)
PEG 2000	1.22 ^M^	1800–2200 ^V^	52 ^V^	n.a.
PEG 4000	1.22 ^M^	3500–4500 ^V^	58 ^V^	n.a.
PEG 6000	1.22 ^M^	5000–7000 ^V^	60 ^V^	n.a.
PEG 8000	1.22 ^M^	7300–9000 ^V^	60 ^V^	n.a.
PEG 20,000	1.22 ^M^	20,000 ^V^	60 ^V^	n.a.
PVB 30 H	1.11 ^M^	32,000–35,000 ^V^	68 ^V^	206 ^M^
PMMA Degalan 7 E	1.18 ^M^	159,000 ^M^	96–109 ^M^	240 ^M^
Stearic acid	0.92 ^M^	284 ^V^	69–85 ^V^	170 ^M^
PAT-77 P	0.97 ^M^	n.a.	85–95 ^V^	n.a.
PAT-659/CB	0.96 ^M^	n.a	45–75 ^V^	n.a.

^M^: measured data, ^V^: vendor’s data sheet.

**Table 2:** Extruded PEG/PVB filaments.

Feedstock	Extrusion Temperature (°C)	Filament Diameter (mm)
PEG 2000/PVB	90	2.82 ± 0.02
PEG 8000/PVB	90	2.80 ± 0.01
PEG 20,000/PVB	90	2.86 ± 0.01

**Table 3:** FFF printing parameters for different PEG/PVB systems (solid load: 60 vol%, 5 wt% SA as surfactant).

FFF Printing Parameter	PEG 2000/PVB	PEG 8000/PVB	PEG 20,000/PVB
Temperature (°C)	230	230	180
Platform temperature (°C)	55	55	60
Printing speed (mm/s)	5	5	5
Printing speed first layer (mm/s)	5	5	2.5
Nozzle diameter (mm)	0.3	0.3	0.3
Infill (%)	105	105	105
Overlap contour lines with infill (mm)	0.2	0.2	0.2

**Table 4:** Extruded PEG(8000)60/PMMA40 filaments (solid load 60 vol%).

Feedstock	Extrusion Temperature (°C)	Filament Diameter (mm)
SA	106	2.81 ± 0.04
PAT77P	70	2.87 ± 0.02
PAT659/CB	n.a.	n.a.

**Table 5:** FFF and FFD printing parameters for the selected PEG (8000)60/PMMA40 applying both PAT surfactants (solid load 60 vol%).

Printing Parameter	FFF (X350Pro)	FFD (150H)
Temperature (°C)	210	220
Platform temperature (°C)	50	60
Printing speed (mm/s)	5–10	5
Printing speed first layer (mm/s)	3	3
Nozzle diameter (mm)	0.15/0.3/0.4	0.4
Infill (%)	105	105
Overlap contour lines with infill (mm)	0.2	0.2

## Data Availability

Not applicable.
